# District-level analysis of socio-demographic factors and COVID-19 infections in Greater Accra and Ashanti regions, Ghana

**DOI:** 10.3389/fpubh.2023.1140108

**Published:** 2023-04-13

**Authors:** Alex Barimah Owusu, Gerald Albert Baeribameng Yiran, Seth K. Afagbedzi, Edwin Takyi

**Affiliations:** ^1^Remote Sensing and GIS Applications Laboratory, Department of Geography and Resource Development, University of Ghana, Legon, Ghana; ^2^Department of Biostatistics, School of Public Health, University of Ghana, Legon, Ghana

**Keywords:** COVID-19 infections, socio-demographic factors, religion, ethnicity, greater

## Abstract

Since December 2019 when COVID-19 was detected, it took the world by surprise in terms of spread and morbidity/mortality. The high rate of spread and casualties recorded from COVID-19 called for research in all directions to find ways to contain and reverse the incidences. It is against this background that this paper sought to measure the association of the socio-demographic factors in the hard-hit districts in Greater Accra and Ashanti to analyze its relationship with the novel COVID-19 virus. Data on COVID-19 cases from 35 Districts in both Greater Accra and Ashanti Regions were collected from the Ghana Health Service and population data from Ghana Statistical Service. Descriptive statistics and regression analysis were generated using R. We found that some socio-demographic variables have an association with COVID-19 infections. For example, age and religion especially Christianity and Islam pose risk to COVID-19. The population aged 15–64 was particularly at high risk of infections due to the high level of movement of this age group. We, therefore, recommend that places of congregation such as Churches and Mosques be targeted for vigorous sensitization on COVID-19 protocols and prevention. Also, districts with a high population between the ages of 15–64 should step sensitization efforts to educate their inhabitants on the need to reduce travel and related activities to curb the spread of the virus.

## 1. Background

It was on the 31st of December 2019 that news of cases of a disease like pneumonia whose antilogy was not known at the time detected in Wuhan city of China emerged (1). The World Health Organization (WHO) announced on January 12, 2020 that a novel coronavirus was the source of respiratory disease in a group of patients in Wuhan City, Hubei Province, China (2, 3). The disease was given the name COVID-19, and the pathogen was identified as SARS-Coronavirus-2 (an RNA virus) (SARS-CoV-2) (4).

The virus is spread mostly by contact with minute droplets produced by an infected person coughing, sneezing, or talking (5, 6). While a large percentage of infected people are asymptomatic, fever, cough, acute respiratory distress, lethargy, and failure to clear after 3 to 5 days of antibiotic treatment are the most prevalent symptoms in clinical cases. Secondary outcomes include the incidence of pneumonia and acute respiratory distress syndrome which has the resultant effect of organ function damage, including acute kidney injury, cardiac injury, liver dysfunction, and a host of other complications that required patients to be put on mechanical ventilation (3, 7).

Before the confirmation of the epidemic in Ghana, the National Disease Surveillance Department of the Ghana Health Service conducted a readiness assessment and developed a response strategy (1). Furthermore, the country provided orientation at the Kotoka International Airport (KIA) and other ports of entry for effective screening and handling of suspected cases, as well as contact tracing training for Alumni and Residents of the Ghana Field Epidemiology and Laboratory Training Program (GFELTP) and Ghana Health Service staff (GHS).

Ghana’s health ministry announced the first two cases of COVID-19 on March 12, 2020 (1). As a first response, all public meetings such as religious gatherings and festivals were prohibited on March 15. On March 16th, a ban on all public gatherings, as well as the closure of schools, churches, mosques, and other places of worship were announced. On March 17, a ban on entry for travelers arriving from a country with more than 200 confirmed COVID-19 cases within the previous 14 days was also announced. The government also announced a mandatory quarantine of all travelers arriving 48 h before the closure of the country’s borders. On the 30th of March, a partial lockout was implemented in areas known as “hotspots” for public safety reasons. These “hotspot” areas were in two regions, which are the Greater Accra and the Ashanti region.

On April 20th, the limitations on Accra and Kumasi were eased, and on April 26th, the usage of face masks became necessary. Even though the lockdown was lifted after three weeks, post-lockdown procedures were implemented to keep the illness from spreading. Early in the COVID-19 pandemic, Ghana’s response was hailed as one of the best among African countries; its innovative testing approach and science-driven political leadership (8, 9). The country’s COVID-19 outbreak response was diverse, contact tracing capacity was strengthened by training several surveillance officers and more treatment centers and Intensive Care Unit beds were established to handle cases (8). There was a provision of psychosocial support and protective gear to several persons. Personal hygiene and self-protection measures were strictly enforced, and these include wearing a nose mask, restriction on social gatherings, social separation, an increase in the number of testing stations, and humanitarian aid for Ghanaians were among them. As the nation continues to increase surveillance and other response actions, this has become the new normal (10).

The impact of the COVID-19 pandemic has been phenomenal. There has been an unprecedented challenge to public health, world of work, and food security (11, 12). The social, demographic, and economic disruption caused by the pandemic is devastating and millions of people worldwide are at risk of falling into extreme poverty, job losses, shuttered businesses, and gaps in schooling, to violence and addiction, among others (13, 14). The consequences of this disease are no different in Ghana, especially in the epicenters such as Greater Accra and Ashanti regions. Because of this, this paper sought to measure the strength of the association between socio-demographic factors and the increase in COVID-19 virus infections in the hard-hit districts in Greater Accra and Ashanti regions, Ghana.

The need for this analysis is to determine the strength of socio-demographic variables in contributing to the increased number of cases in such “hotspot” areas. With such information, the government can easily develop and implement the exact measures to minimize the increased number of cases in these hotspot areas. This can help in the allocation of resources such as the formation of quarantine sites, distributing hand sanitizers and nose masks, and the building of COVID-19 facilities to help stop the spread of the virus.

### 1.1. Impact of COVID-19

Since the 1918 influenza pandemic, the novel human coronavirus has been responsible for five (5) pandemics, including the COVID-19 outbreak (15). The COVID-19 began in Wuhan, China, and quickly spread throughout the world. On January 2020, the World Health Organization (WHO) named the new virus the 2019 novel coronavirus (2019 nCoV), and on February 12, 2020, it was renamed the infectious coronavirus disease 2019 (COVID-19) (16). On March 11, 2020, the WHO declared COVID-19 to be a pandemic.

From its outbreak in December 2019 up to February 2023, an estimate of about 676,208,868 recorded case, 648,612,009 recoveries and 6,771,722 million deaths have been recorded (17). For the same period in Ghana, 171,112 cases, 14 active, 2 serious, 169,636 recoveries, and 1,462 deaths had been documented (17). COVID-19 Pandemic has had great impact on the worldwide population, including multiple deaths and economic hardship (15). COVID-19 as a pandemic has affected a number of regions globally, apart from China and Thailand who recorded the initial cases (16). Like any other continent, Africa has had its fair share of the infections and continues to spread throughout the continent. In fact, the continent was considered as vulnerable due to the swiftly rate at which the pandemic was spreading (18). On the 14th of February 2020, Egypt confirmed its first case of COVID-19, while the first case from Sub-Saharan Africa was recorded in Nigeria on the 27th of February 2020, (18). After the first cases reported on march 12, MOH, in conjunction with the GHS began tracing the people suspected to have had contact with cases, particularly those returning from outside the country upon their arrival at the entry points and also a contact tracing activity on all those suspected having sufficient with the two suspected cases (19).

Due to the rate at which COVID-19 pandemic spread across the continents, the United Nations Framework (2020) reported COVID-19 pandemic as the worst recorded with the highest historic levels of unemployment, restrictions on people’s freedom of movement, and heightened levels of hardship in human history (20).

The COVID-19 pandemic has had severe humanitarian consequences such that an attempt to estimate the overall cost in human life, is considered the unthinkable, although the impacts are still being determined around the world in terms of political, social, economic, and health systems networks and education. Aside the high cost of life and a severe health crisis, the world is experiencing an economic downturn that is having a significant influence on the well-being of huge segments of the world’s population (21). According to UNIDO (2020) the most concerning consequences of COVID-19 worldwide pandemic, beyond human life is felt in terms of the economic damage it has caused around the world. Expectations for global economic development were all shattered as a result of investment setbacks (22). All sectors of economics have been devastated resulting in Global Gross Domestic Product fallen by over 78%, and in some regions experiencing negative growth from the 3.2% before the COVID-19 pandemic to 1.8% during the countries’ lockdown limitations (UNIDO, 2020). High levels of supply shortages have disturbed global supply systems, resulting in soaring prices (23). The ILO (24) provides a full assessment of the impact of the pandemic on employment dynamics; the findings show that unemployment and under-employment have skyrocketed. Unemployment has risen by 5.3, 13.0, and 24.7 million, considering low, mild, and high impact scenarios, respectively. The global financial crisis increased unemployment by 22 million, implying that in a high impact scenario, the pandemic has a deeper consequence. Downward wage and working hours’ adjustments are worsening the under-employment. The number of hours worked has plummeted even far more than the situation in the 2008 global financial crisis. In Ghana, the COVID-19 mitigation measures have had an unmeasurable impact on Government, business community and individuals well beyond the human life lost. Impacts are seen in terms of strained government budget and liquidity constraints, increased unemployment rate, a decline in income generation, and disruption in transportation, among other essential services (25). Ghana Statistical Service in its industrial survey conducted and published in 2021 attested to the devastating impact of COVID-19 on Ghanaian businesses. They indicated that 35.7% of business establishments had to close during the partial lockdown, 46.1% of business reduced wages for 25.7% of the workforce (about 770,124 workers) (26).

## 2. Materials and methods

### 2.1. Study area

**Figure 1:**
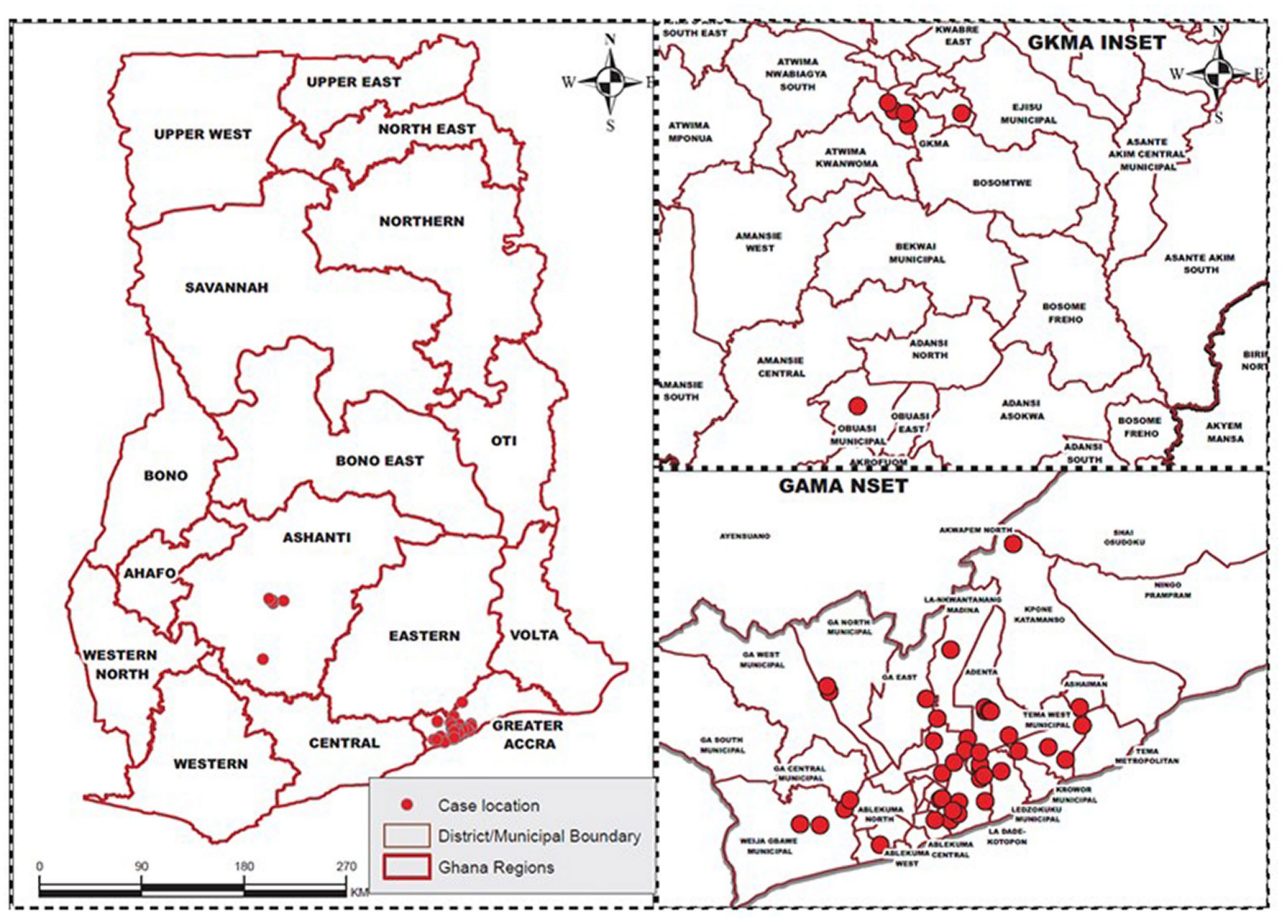
Study area.

The study considered districts from two regions in Ghana that is; Greater Accra and Ashanti region which were the epicenters of COVID-19 during the early stages of the disease in Ghana and districts from the two regions were studied (see Figure 1). The location, socio-demographic, economic, and health profiles of the two regions, Greater Accra and Ashanti regions are discussed below. First the Greater Accra Region. On July 23, 1982, the Greater Accra Region, which was previously joined to the Eastern Region, was geographically and legally separated. It is one of the Ghana’s sixteen (22) administrative regions. It is located in the country’s southern coast, bounded by Eastern Region located at the north, at the east of Greater Accra is the Volta Region, the Central Region located at its western zone, and the Gulf of Guinea at the south. It covers a land area of 3, 245 km^2^, which is 1.4% of the total land area in Ghana.

Greater Accra Region includes Ghana’s capital city, Accra, as well as 28 Metropolitan, Municipal and District Assemblies (MMDAs). The region is made up of four district assemblies, 23 municipal assemblies, and two metropolitan assemblies. Each of the MMDAs is led by a Chief Executive.

Despite its small land surface area, the Greater Accra region is the most densely populated. With a population of 5,455,692, the region’s female population (2,776,629) outnumbers the male population (2,679,063) (27). More than half of the population, 3,295,777 people, are under the age of 65 and from the working class. The region has become home to a diverse range of groups and ethnicities from across the country, with the Ga-Dangme people serving as the primary indigenous group. The Akan are the largest ethnic group in the Greater Accra Region, followed by Ga–Dangme and the Ewes. Because of in-migration from the country’s northern regions, the region has a very high population density and growth rate. Even though international migration is low, migrants from other African countries into the region outnumber those from outside the African continent. The most populous district is the Accra Metropolitan Assembly, followed by the Tema Metropolitan Assembly. Greater Accra Region has the highest literacy rate among the 16 administrative regions, at 87% for both urban and rural male and female populations.

The region’s population of 5,455,692 demonstrates that it is economically active. More than half of the economically active populace (51.8%) are self-employed, and 32.6% are employed by someone else. Men are 1.5 times more likely to be employed than women. The region is made up of sales and general workers who are typically concentrated in the two metropolitan areas. Agriculture, hunting, fish farming, and animal husbandry employ roughly half of Greater Accra’s population. Salt mining is the region’s primary mining activity. On the other hand, approximately 11.3% of the Greater Accra population is unemployed (27). Accra, the country’s capital, is home to much of the country’s infrastructure and social amenities. With several national highways connecting population areas throughout the country, the city is also home to many corporate headquarters and Ministry offices.

The Ashanti Region is located in Ghana’s middle belt, covering a total land surface area of 24,389 km^2^. It is situated between longitudes 0.15 W and 2.25 W and latitudes 5.50 N and 7.46 N. It is bordered by five administrative regions: the Eastern Region located to the east, the Western North Region facing the south-west, the Central Region (to the south), the Bono East Region (to the north), Ahafo Region (to the east), and Bono Region located at the North-West. The Ashanti region is divided into 43 sub-divisions, 24 of which are district assemblies, 18 of which are municipal district assemblies, and one metropolitan assembly, the Kumasi Metropolitan Assembly. Kumasi is the regional capital.

After Greater Accra Region, Ashanti Region has the second largest population. The population increased to 5,440,463 people in the year 2021, according to the Ghana Statistical Service. Females represented 50.7% of the populace (2,760,549), while males amounted to 49.3% (2,679,914). Ashanti region, like the Greater Accra region, has a youthful population and many working-class residents. Between 2010 and 2020, the population increased at a rate of 1.2 percent. Over half of the region’s populace lives in urban zones, making it the second most densely populated after the Greater Accra Region. The region has a high rate of immigration, while others migrate to Western and African countries. The Ashantis are the main indigenes of the Ashanti Region, but other ethnic groups live there as well, as in other regions. The Asante nation’s social administration is led by traditional chiefs and elders, and each division has its chief or paramount chief.

Forestry and agriculture (the production of timber and livestock) are the region’s most important economic activities. Cocoa is an important crop in some parts of the region, and the region also has the country’s largest mining site. Furthermore, other residents provide other services such as food, lodging, manufacturing, retail, and wholesale. According to the National Population Council’s 2018 report, Ashanti Region had the highest employment rate for both males and females, at 19.7% and 18.8%, respectively. Meanwhile, the region had the second-highest unemployment rate (10.3%) (27).

### 2.2. Health profile and COVID-19 response

The novel coronavirus was confirmed a worldwide pandemic by The World Health Organization (WHO), on 11th March 2020. With a wide range of mild symptoms to severe illness, the virus could cause fever, shortness of breath, headaches, loss of smell and taste, and sore throat; just to mention a few (Center for Disease Control and Prevention, 2021). The first two confirmed cases of COVID-19 in Ghana were first diagnosed in the Greater Accra Region on 12th March 2020 (28). Since then, the global pandemic had increased gradually in all regions with higher cases in the Greater Accra region and the Ashanti Region.

Between 30th March and 20th April 2020, the strictest lockdowns were witnessed in the most populated cities in the regions, Greater Accra Metropolitan Area (GAMA), Greater Accra Region, and the Greater Kumasi Metropolitan Area and Contiguous Districts (GKMA) in Ashanti region. There was a closure of all activities in these two regions whiles essential services were being provided in Accra and Kumasi. Initiated by the Government of Ghana, the contact tracing system was adopted when the first cases were confirmed. The contact tracing system was a key strategy used to strengthen protection and detect early infections. Within a month, the number of cases recorded increased by more than 50%; 2,655 to 4,131 from 18,000 contacts (10). Initially, only two health facilities were able to test for the virus: the Kumasi Centre for Collaborative Research in Tropical Medicine (KCCR) and the Noguchi Memorial Institute for Medical Research (NMIMR) (29). Drones were also employed by the Ministry of Health to gather COVID-19 samples from over 1,000 health locations. Affected people were isolated and monitored by health workers. According to the government, contact tracing and strict protocols aided in lowering the virus’s causality rate. In the Ashanti region, Metropolitan, Municipal, and District assemblies were required to enforce strict safety protocols, and violators were arrested and turned over to authorities.

The COVAX Facility delivered 600,000 AstraZeneca COVID-19 vaccinations to Ghana, making it the first country to do so. The vaccines were distributed to selected health facilities in Greater Accra’s 25 districts and Ashanti’s 16 districts (30). About 325 vaccination sites were set up in the 25 districts of the Greater Accra Region. By the end of the first week of March 2021, a total of 104,174 people were in Greater Accra and as of 31st March, 555,259 doses of vaccines had been administered nationwide (31).

The rate of increase in infected people has recently slowed. The total number of confirmed cases is 155,665, with Greater Accra leading the way with 86, 732 cases, followed by Ashanti Region with 22, 253 cases (32). The authorities have scheduled the distribution of vaccines for the health facilities in the various districts to ensure equitable distribution. Total vaccine doses administered as of 17th January 2022 were 9,342,953 (33). The Ghana Health Service continues to schedule vaccination times whiles companies and individuals adhere to safety protocols. In December 2021, a vaccination drive was held and was declared mandatory for all health workers and public servants. Seven million more doses were delivered in the country by the end of December 2021.

In Ghana, one of the most pressing issues is health. Since independence, the country’s population growth, particularly in Greater Accra and Ashanti regions, has been viewed as unplanned, with implications on public health. Numerous health centers, led by the Ministry of Health and the Ghana Health Service, have been established in the regions to address the numerous health issues that have arisen. Significant efforts have been made in policy development, guidelines development, and in-service training.

Despite these advancements, the state of health and health care in Ghana varies. While urban areas are well served by health facilities that are available in all areas, rural areas frequently struggle with availability. In the worst-case scenario, modern health care services are unavailable. Although there are over 1,500 health centers, some communities still lack access to health centers and staff. Per the standard of the World Health Organization, doctor to patient ratio is 1:600. Yet, Ghana’s status remains 1: 10,000 for a doctor to patients and 1: 9,000 for the nurse to patients. In addition, there is less than one bed per 1,000 people (34). With these findings, the country faces challenges in terms of facilities and workforce; any additional increase in the patient ratio will put a strain on the health sector.

In the early days of COVID-19 (as of July 2020), over 2,000 health workers were infected with COVID-19, with 6 confirmed dead (33). In other news, the Ghana Health Service announced the closure of 99 health facilities in the Ashanti Region in January 2022 due to an increase in the Omicron variant of COVID-19 (35). The Omicron variant of the COVID-19 outbreak overburdened healthcare facilities because staff and healthcare workers became infected with the virus, reducing the limited workforce.

Not only did COVID-19 have a negative impact on healthcare, but it also created opportunities for further improvement in public and individual health. Following the discovery of COVID-19, a lot of focus went to the health industry and individual health. New facilities and health centers were constructed, and others were planned. In the Greater Accra region, extra ICU beds were built, as well as a treatment center in the Ashanti region. Agenda 111 was a government of Ghana program to build 101 health facilities across the country that came forth as a result of the pandemic. In addition, four distinct forms of incentives were given to health personnel to compensate them for their hard work and stressful activities.

Ghanaians, particularly those in Greater Accra and the Ashanti Region, became more aware of personal hygiene, while some thought the whole thing was a “scam.” In Greater Accra, audio communication regarding personal hygiene and hand washing was made in some transport stations, and open areas. Even though the number of regular patients in hospitals has decreased due to the fear of being admitted as a COVID-19 patient, residents have made an effort to improve their health by adhering to all protocols and focusing on self-care.

COVID-19 sparked the imagination of the entire human race to respond to disasters. Ghana, as a country, is constantly updating its protocols and combating solutions. On the bright side, new developments in health and human development will continue to emerge.

### 2.3. Data and method of data analysis

#### 2.3.1. Data

The study made use of District-level COVID-19 secondary data from the Ghana Health Service and population data from the Ghana Statistical Service. The data consist of 35 observations, which are the number of districts in the two regions with recorded COVID-19 cases. Districts with no COVID-19 cases were excluded. The data cleaning and preparations were done using R programming language. Due to the large variability in the independent variable, the log transformation was used to reduce variation in the observations. The multiple linear assumptions were tested for the study. These are:

1. A linear relationship between the dependent variable (the number of COVID-19 cases) and the levels of each independent variable (socio-demographic factors)
2. The independent variables (socio-demographic factors) are not highly correlated with each other
3. The variance of the residual is constant
4. The residuals are normally distributed.

#### 2.3.2. Data analysis

This is a descriptive cross-sectional study using a quantitative approach. The study made use of secondary District-level COVID-19 data from Ghana Health Service from March 2020 to September 2021 and population data from Ghana Statistical Service, compiled from 2010 PHC.

Multiple linear regression was used to examine the relationship between the levels of each socio-demographic factor and COVID-19 infection at the district level at a 5% level of statistical significance. The dependent variable (number of COVID-19 cases) is numerical and the independent - (socio-demographic factors) considered in the study are age with three levels (Under 15, 15–64, 65+), ethnic groups (Akan, Ga-Dangme, and Gurma) and religious groups (Christianity, Islam, and Traditionalist). Due to the high variability in the data obtained, the log transformation was used to reduce variation in the data and to normalize the data. Taking the log of the variable will effectively change the base from a unit change to a percentage change. However, the data had no missing values. (Due to collinearity between the independent variables, an estimate from overall multiple linear regression was not appropriate, we, therefore, analyze the effect of COVID-19 on each of the socio-demographic factors to see the relationship with each of them separately.)

The descriptive analysis and regression analysis was carried out using R. The hypothesis for the multiple linear regression is as follows.

*H0*: There is no significant relationship between COVID-19 and the levels of each of the socio-demographic factors.

*H1*: There is a significant relationship between COVID-19 and the levels of each of the socio-demographic factors.

## 3. Results

As shown in Table 1, the variable ln (Under 15) possesses a negative coefficient indicating that a 1% increase in the number of Under 15 aged persons is expected to decrease COVID cases by 40.41%. In the case of the variable ln (15–64), the coefficient is positive which indicates that 1% increase in the number of 15–64 aged persons in a district is expected to increase COVID cases by 34.27%. The variable ln (64+) with a positive coefficient indicates that a 1% increase in the number of 64+ aged persons is expected to increase COVID-19 cases by 19.57%. There are statistical significances for all levels of the Age factor at a 95% confidence level, hence the number of cases is associated with the age profile of a district. However, Ethnicity had much lower coefficients as can be seen in Table 2.

**Table 1:** Regressing COVID on ln (age).

Age	Coefficient	Std. err.	*t*	*p* > |*t*|	[95% conf. interval]
ln (Under 15)	−4040.80	1669.70	−2.42	0.022	[−7446.19, −635.42]
ln (15–64)	3427.45	996.06	3.44	0.002	[1395.97, 5458.93]
ln (65+)	1956.55	784.33	2.49	0.018	[356.90, 3556.19]
Constant	−10806.80	3411.98	−3.17	0.003	[−17765.58, −3848.02]

**Table 2:** Regressing COVID on ln (ethnicity).

Ethnicity	Coefficient	Std. err.	*t*	*p* > |*t*|	[95% conf. interval]
ln (Akan)	742.52	208.21	3.57	0.001	[317.87, 1167.17]
ln (Ga-Dangme)	323.73	78.58	4.12	0.000	[163.48, 483.99]
ln (Gurma)	435.98	172.78	2.52	0.017	[83.60, 788.37]
Constant	−13551.91	2107.05	−6.43	0.000	[−17849.27, −9254.55]

In Table 2, the variable ln (Akan) with a positive coefficient of 742.52 indicates that a 1% increase in the number of Akans is expected to increase COVID-19 cases by 7.43. The variable ln (Ga-Dangme) with a positive coefficient indicates that a 1% increase in the number of Ga-Dangme is expected to increase COVID cases by 3.24. The variable ln (Gurma) with a positive coefficient indicates that a 1% increase in the number of Gurma is expected to increase COVID cases by 4.36. However, there are statistical significances for all levels of Ethnicity at a 95% confidence level. According to the GSS (2013), the population of Ghana is youthful, that is, a larger percentage of the population is in the youth category. Thus, age is a strong factor that explains the low cases and fatalities in Ghana and other countries where the majority of the population is young. However, the implication of this is that work and productivity output will be affected as all the people in the working class are in the affected age category. Religion returned slightly higher coefficients compared to ethnicity (Table 3).

**Table 3:** Regressing COVID on ln (religion).

Religion	Coefficient	Std. err.	*t*	*p* > |*t*|	[95% conf. interval]
ln (Christian)	1192.63	292.43	4.08	0.000	[596.22, 1789.03]
ln (Islam)	575.07	264.92	2.17	0.038	[34.75, 1115.38]
ln (traditionalist)	−173.05	227.39	−0.76	0.452	[−636.82, 290.71]
Constant	−17355.36	2359.32	−7.36	0.000	[−22167.23, −12543.49]

The variable In (Christians) with a positive coefficient indicates that a 1% increase in the number of Christians is expected to increase COVID-19 cases by 11.93. The variable In (Islam) with a positive coefficient indicates that a 1% increase in the number of Islam is expected to increase COVID-19 cases by 5.75. The variable ln (Traditionalist) with a negative coefficient indicates that a 1% increase in the number of Traditionalists is expected to decrease COVID-19 cases by 1.73. However, there are statistical significances for Christianity and Islam while Traditionalist is not statistically significant at a 95% confidence level. This means that circular religion had more effect than traditional religion. This is so because these religions (Islam and Christianity) require their believers to gather in large numbers to worship and thus exposing the people to the pandemic. Although religious gatherings were banned (36), people still gathered to pray once they never exceeded 25. Although COVID-19 protocols were strictly enforced within the church auditoriums and mosques, these were not strictly observed after the people were outside. Another observation was that the ban on social gatherings was not obeyed especially during funerals which were largely religious gatherings. These increase the risk of contracting the disease.

## 4. Discussions and recommendations

This study provides a comprehensive study of the socio-demographic risk factors of COVID-19, using data for the two most populated regions in Ghana. From the analysis, we found out that districts with a high number of Christians and Muslims are likely to record a high number of COVID-19 cases. In an article by Wildman et al. (37) it was found that, as of the end of the first week of March 2020, almost two-thirds of coronavirus infections (nearly 5,000 cases) were traced back to Patient 31, an individual who worshipped at Shincheonji Church of Jesus in Daegu at South Korea. This goes in line with the analysis in this result that Christianity and Islam which have more religious activity are major factors in the spread of the COVID-19 virus.

However, considering the socio-demographic factor such as age, it was found that the adult regressor was statistically significant with a positive coefficient. This is because they form a large part of the working class and engage in more activities which lead to the spread of the virus. During the period, schools were closed, and children were more at home with fewer outdoor activities, but the adult population had to move from one place to another largely for household shopping, international travel, and a few social gatherings that were permitted (38–40). Thus, the adult population had more chances of coming into contact with the virus than the children. This finding is further strengthened as we found that above 65 years (Table 3), the coefficient decreases to almost half of the age bracket of 15 to 65 indicating fewer infections among people older than 65. This is not surprising as children and adults above 65 are reported to be less susceptible to infectious diseases (40). However, if serious restrictions are to be enforced, it could further worsen the already ailing economies of these poor countries. This is because the working class is within this age group and productivity will be brought to its lowest level and thus unable to sustain the economies. Many studies on the impact of COVID-19 on household livelihoods around the world indicate that the majority of households are not able to recover to pre-COVID levels (32, 38, 41). This, therefore, calls for a balanced approach to dealing with the pandemic.

We conclude that some socio-demographic variables have an association with coronavirus infections. For example, age and religion, especially Christianity and Islam, pose a risk of the coronavirus. We, therefore, recommend that places of the congregation such as Churches and Mosques be targeted for vigorous sensitization on COVID-19 protocols and prevention. Also, districts with a high population of the age of 15–64 should be educated on the spread of the virus and the need to reduce travel and related activities to decrease the number of cases in such districts.

## 5. Limitation

This study is more ecological and hence comes with the limitation of ecological design. Also, only three demographic characteristics were considered in the study hence the careful interpretation of results from this study should be done.

## Data availability statement

The original contributions presented in the study are included in the article/Supplementary material, further inquiries can be directed to the corresponding author.

## Author contributions

SA contributed to the writing and review of the manuscript. AO designed the study and supported the overall research procedure and contributed to the writing of the manuscript. GY contributed to the writing and review of the manuscript. ET conceived collected data, and performed statistical analysis. All authors contributed to the article and approved the submitted version.

## Conflict of interest

The authors declare that the research was conducted in the absence of any commercial or financial relationships that could be construed as a potential conflict of interest.

## Publisher’s note

All claims expressed in this article are solely those of the authors and do not necessarily represent those of their affiliated organizations, or those of the publisher, the editors and the reviewers. Any product that may be evaluated in this article, or claim that may be made by its manufacturer, is not guaranteed or endorsed by the publisher.
