# Supplementary material for: District-level analysis of socio-demographic factors and COVID-19 infections in Greater Accra and Ashanti regions, Ghana
Source: Front Public Health. 2023 Apr 13;11:1140108. doi: 10.3389/fpubh.2023.1140108 (PMC10133534; doi:10.3389/fpubh.2023.1140108)

Appendix

The figure below is a correlation matrix used to measure the relationship between the dependent and independent variable.


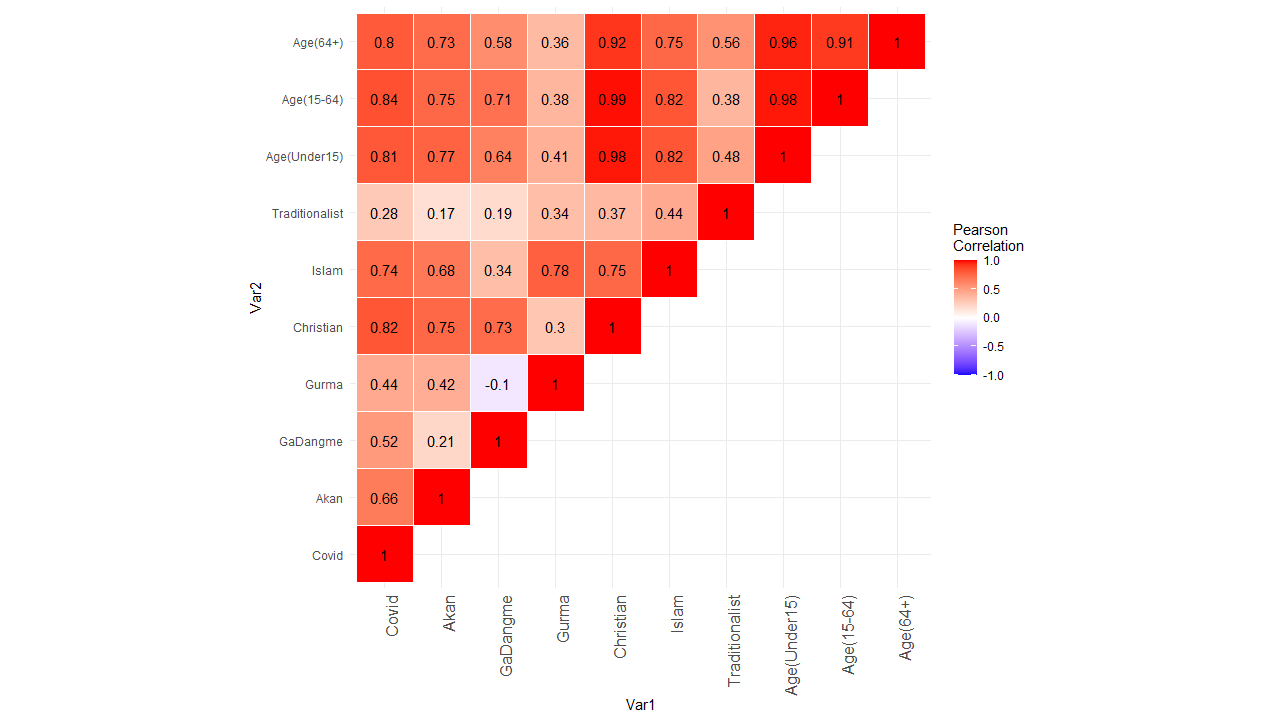

Supplement: Supplementary file 1 [file Data_Sheet_1.docx]
